# Changes in the Immunology of Breast Milk From Obese or Overweight Women: a Brief Review

**DOI:** 10.7759/cureus.52207

**Published:** 2024-01-13

**Authors:** Jorge I Zurutuza, Santiago Gonzalez, Ana L Calderón, Mario Caba, Fernando R Ramos

**Affiliations:** 1 Epidemiology and Biostatistics, Centro de Investigaciones Biomedicas, Universidad Veracruzana, Xalapa, MEX; 2 Chemistry, Centro de Investigaciones Biomédicas, Universidad Veracruzana, Xalapa, MEX; 3 Neuropathology, Instituto Nacional de Neurología y Neurocirugia, Ciudad de Mexico, MEX; 4 Neuroscience, Centro de Investigaciones Biomédicas, Universidad Veracruzana, Xalapa, MEX; 5 Chemistry, Instituto de Quimica Aplicada, Universidad Veracruzana, Xalapa, MEX

**Keywords:** overweight, human milk, immunology, obesity, breastfeeding

## Abstract

A systematic search was carried out through search platforms and specialized databases, such as Academic Google, PubMed, and Scopus, using thesauri: breast feeding, obesity, immunology, and human milk in English and Spanish, and those articles published from January 2000 to December 2021, in both languages. Only those reports that included quantitative data on immunological components in the milk of normal-weight and overweight women were considered. The PRISMA 2020 guides were used, and a total of 306 articles were reviewed, of which a total of 33 were included, according to the basic inclusion criteria. It was observed that in obese mothers, there is an increase in certain immune cells, such as B lymphocytes, cytotoxic T lymphocytes, and NK cells, and cytokines, such as IL-6 and IFN-γ; other alterations included the bacterial population and proteins with antibacterial action. Also, a decrease in growth factors such as TGF-β and IFG-1 was documented in overweight women. Immunoglobulin concentrations did not show substantial changes. This brief review shows that maternal overweight is associated with changes in the biochemical and immunological parameters of milk.

## Introduction and background

Introduction

Breastfeeding provides a wide range of benefits for the newborn that go beyond those related to nutrition [1]. Benefits have been found in relation to the intestinal microbiota [2], the immune response [3], epigenetic effects [4], and survival in preterm newborns [5] and even new therapeutic applications, such as an effective treatment for diaper rash, atopic eczema, and diaper dermatitis [6]. It is known that breast milk has a large number of nutritional components and a great dynamic capacity for adaptation [7], since it is capable of changing according to the needs of the newborn, as well as the characteristics of the mother and the environment, in which both develop, and this includes exposure to environmental antigens [8].

In the 1980s, it was thought that the primary benefit of breast milk was to promote the growth and development of the newborn [9]. Thereafter, the relevance of the immunological components was described, when comparing susceptibility to infectious diseases and mortality due to them in infants fed at the mother’s breast versus infants who received substitutes or milk from other animals [10,11]. The functions of breast milk in the new paradigm were increased, from simply providing nutrients, to achieving the survival of the newborn in a hostile environment full of pathogens and antigenic stimuli, and to contributing to the acquisition of the adequate natural microbiota, with which the infant will coexist for the rest of his life. This bacterial set must be recognized as its own, and changes in breast milk will contribute to the immune training of the newborn [12].

This ability to modify its composition according to the environment suggests that it can also change in pathological states, especially those that alter the balance in the immune response, favoring inflammation. Therefore, changes caused by highly prevalent diseases, such as obesity, diabetes mellitus, and hypertension, and their long-term complications, such as cardiovascular or kidney disease, should be studied. These pathologies were the first causes of death worldwide in 2019, according to the World Health Organization (WHO), regardless of the socioeconomic level of the country in question [13].

Obesity is a pro-inflammatory state that is associated with multiple chronic degenerative diseases [14] and that can by itself aggravate infectious diseases such as SARS-CoV-2 [15]. According to the WHO, the global prevalence in 2016 was 13% [13]; however, in Mexico, the prevalence in women older than 20 years is 40.2% [16], precisely in the reproductive stage. This requires the study of possible modifications or effects on the immunological characteristics of breast milk.

Breast milk is the ideal food for newborns; therefore, the objective of this work was to investigate, through a systematic review of the literature, whether overweight and obesity affect the composition and quality of this milk, especially in the immunological component and what repercussions these changes can have on the infant.

Before entering into the parameters that are altered in breast milk in obese and non-obese people, it is necessary to know about the generalities of the composition of breast milk. Breast milk includes cells, nutrients, and various chemical compounds. An important part of its composition is the nitrogen component, which has two fractions: protein nitrogen (75% of total nitrogen) and non-protein nitrogen (25% of total). The protein nitrogen component includes casein and whey proteins in a 40:60 ratio. Casein is mainly made up of the β-casein subunit. Of the whey proteins, α-lactalbumin is the most abundant and is rich in cysteine and tryptophan. Lactoferrin, also a whey protein, binds two iron atoms and competes with some bacteria in the baby’s intestinal tract for this metal, which is essential for their growth and also is bactericidal. The non-protein nitrogen fraction includes urea, creatinine, creatine, uric acid, free amino acids and ammonia, polyamines, hormones, growth factors, cyclic nucleotides, and nitrogen-containing oligosaccharides. It also includes a large number of amino acids, such as taurine and nucleotides. Taurine deficiency in the early stages has negative effects on retinal development. Nucleotides seem to function as immunomodulators and promoters of bifidobacteria [17]. Immunoglobulins (Ig) are an important component in breast milk, with high concentrations in colostrum. The main one is secretory or dimeric IgA (sIgA) and, to a lesser extent, monomeric IgA, IgM, and IgG. IgA prevents the penetration of pathogens into the intestinal mucosa.

Among the enzymatic components, lysozyme with bactericidal action and lipase stand out, which hydrolyzes fats and releases glycerol and free fatty acids, some of the latter, with antimicrobial activity. Platelet-activating factor acetylhydrolase activates target cells such as platelets and neutrophils by binding to specific G protein-coupled cell surface receptors. It is involved in normal physiological processes such as inflammatory response and hemostasis [18]. With these data in mind, we can analyze the different components of breast milk that are related to immune function.

Lactoferrin

It is one of the glycoproteins integrated into breast milk with a weight of 80 kD [19], which has two iron-binding areas. This protein, which is not degraded even at low pH, competes for iron at the intestinal level and prevents its use by pathogens [20,21]. Values in the general population are 1.5-2.0 g/L in mature milk [22] and 5-6 mg/mL in colostrum [23,24].

Lysozyme

It is a 15 kDa glycoprotein with lytic activity against pathogenic microorganisms, especially against the outer wall of gram-negative bacteria, and inhibition of viral growth (in the free forms) [24]. Its values in the population range between 0.1 and 0.9 g/L [25].

Immunoglobulin A

Ig are proteins capable of recognizing and binding to antigens. IgA is the most abundant in secretions and, therefore, in breast milk [23,26], in which it is mostly found predominantly in dimer form. IgA has neutralization and opsonization functions and is capable of complement activation [27]. The concentration in breast milk is 331 mg/dL (±49.8) in colostrum and 293.1 mg/dL (±47.5) in mature milk [28-30].

Oligosaccharides

These sugars favor the development of the beneficial intestinal microbiota. They also function as immune modulators, control the development of the intestinal epithelium, create a physical barrier in the epithelium, and finally have a direct antimicrobial effect [31]. The concentration is 20-25 g/L in colostrum and 5-20 g/L in mature milk [32].

Cytokines

They are a very broad group of proteins and glycoproteins with functions in the immune and inflammatory response, which first intensify the response and later are responsible for control and downregulation [33]. Cytokines with intense proinflammatory action are interleukin (IL)-1 (its β fraction), IL-2, IL-6, IL-7, IL-8, IL-9, IL-17, interferon-γ (IFN-γ), and tumor necrosis factor-α (TNF-α). Cytokines that favor the control of the immune response and can be described as those with an anti-inflammatory effect are IL-4, IL-10, and IL-13 and transforming growth factor-β (TGF-β) [34]. Colostrum values include a median IL-1β of 2.1 pg/mL, IL-2 of 30.9 pg/mL, IL-4 of 11.8 pg/mL, IL-5 of 73 pg/mL, IL-6 of 301.9 pg/mL, IL-7 of 421.6 pg/mL, IL-8 of 23 157.3 pg/mL, IL-9 of 596.4 pg/mL, IL-10 of 1179, 2 pg/mL, IL-12 of 4.8 pg/mL, IL-13 of 539.4 pg/mL, IL-17 of 309.6 pg/mL, C-reactive protein (CRP) of 0, 38 mg/dL, TNF-α of 4.8 pg/mL, and IFN-γ of 4120.8 pg/mL. For mature milk, the median values include IL-4 of 61 pg/mL, IL-5 of 142 pg/mL, IL-6 of 36.6 pg/mL, IL-7 of 302.6 pg/mL, IL-8 of 1072.7 pg/mL, IL-9 of 252.6 pg/mL, IL-10 of 944.4 pg/mL, IL-13 of 404.3 pg/mL, TNF-α of 4.8 pg/mL, and IFN-γ of 2467.6 pg/mL. Regarding TGF-β, it has a median of 581 pg/mL in colostrum and 177 pg/mL in mature milk [35-37]

Complement

It is a component of the innate immune system, made up of a set of proteins produced by the liver, which cause the lysis of the bacterial wall through the formation of the membrane attack complex or by opsonizing the microorganism [38]. The levels of C3 are 155.6 mg/dL in colostrum and 130.9 mg/dL in mature milk, while they are 47.5 mg/dL in colostrum and 39.4 mg/dL in mature milk for C4 [29].

Immune response cells

Macrophages are the first line of cellular defense of the innate immune response, capable of recognizing antigens present in bacterial walls and ingesting and destroying them. Granulocytes (neutrophils, basophils, and eosinophils) release substances that facilitate the degradation of microorganisms and, in turn, mediate the immune response by increasing it and, finally, the adaptive immune response cells. Cytotoxic T lymphocytes are responsible for the effector activity, while helper T lymphocytes are responsible for controlling the immune response. Finally, B lymphocytes, through the production of antibodies, encompass the adaptive humoral response [39]. The median cell counts in breast milk of term newborns reported in the literature are summarized in Table 1 [40].

**Table 1:** Differences in the median cell counts in human breast milk depending on the type of sample and its alteration in obesity

Cell subpopulation	Colostrum (cells/mL)			Transitional milk (cells/mL)			Mature milk (cells/mL)			Alteration reported in obesity
	Cell count	Range		Cell count	Range		Cell count	Range		
		Lower limit	Upper limit		Lower limit	Upper limit		Lower limit	Upper limit	
CD45^+^ leukocytes	184 000	111 000	291 000	32 450	10 450	66 150	14 700	7 420	56 500	Decreased in colostrum [41]
CD16^+^ monocytes	3 060	903	5 800	364	170	772	307	164	1 160	Decreased in colostrum [41]
CD16^- ^monocytes	1 990	233	5 800	531	89	801	213	28	501	Decreased in colostrum [41]
Neutrophils	23 500	1 198	65 900	5 640	1 221	14 425	2 560	1 490	25 700	Decreased in colostrum [41]
Basophils	2 460	1 490	3 410	435	172	1320	370	96	571	Not reported
Eosinophils	1 760	1 040	152 890	371	75	1 685	269	110	395	Not reported
B lymphocytes	982	333	1 230	149	73	230	59	26	127	Increased in colostrum [42]
Cytotoxic T lymphocytes and NK cells	945	468	1 250	222	66	756	340	128	513	Increased in mature milk [42]
Non-cytotoxic T lymphocytes	5 410	4 640	16 300	1 740	400	5 330	1 290	250	4 040	Not reported
Myeloid precursors	16 900	14 200	21 300	6 295	2 480	11 750	1 490	559	4 560	Not reported
B cell precursors	7 720	1 620	8 370	485	207	1 235	135	74	285	Not reported
Immature granulocytes	12 600	8 410	26 500	2 945	2 160	12 230	2 050	775	14 600	Not reported

Microbiota

Several studies have revealed that colostrum and breast milk are continuous sources of commensal, mutualistic, and potentially probiotic bacteria for the baby’s gut. In the past, human milk from the mother was considered totally sterile. Today, human milk is known to be one of the main sources of bacteria for the infant gut [41]. Suppose a baby consumes approximately 800 mL/day of milk, then the baby ingests approximately between 1 × 10^5^ and 1 × 10^7^ bacteria per day. The predominant bacterial genera found in breast milk are *Streptococcus*, *Staphylococcus*, *Bifidobacterium*, *Propionibacterium*, and *Lactobacillus* [41]. This is very important to know, as the breast milk microbiota contains some of the first microbes to enter the infant’s gut, and depending on the source of the bacteria, different factors could contribute to the milk microbiome. Factors that influence the mother’s gut microbiota, such as obesity or diet, could affect the bacteria that originate in the maternal gut. On the other hand, the mode of delivery, complementary feeding, and breastfeeding mode (directly to the breast or bottled breast milk) could potentially alter the bacteria of both the baby’s oral cavity and the infant’s intestine, thus affecting the development of your immune system [42-44].

## Review

Materials and methods

**Figure 1:**
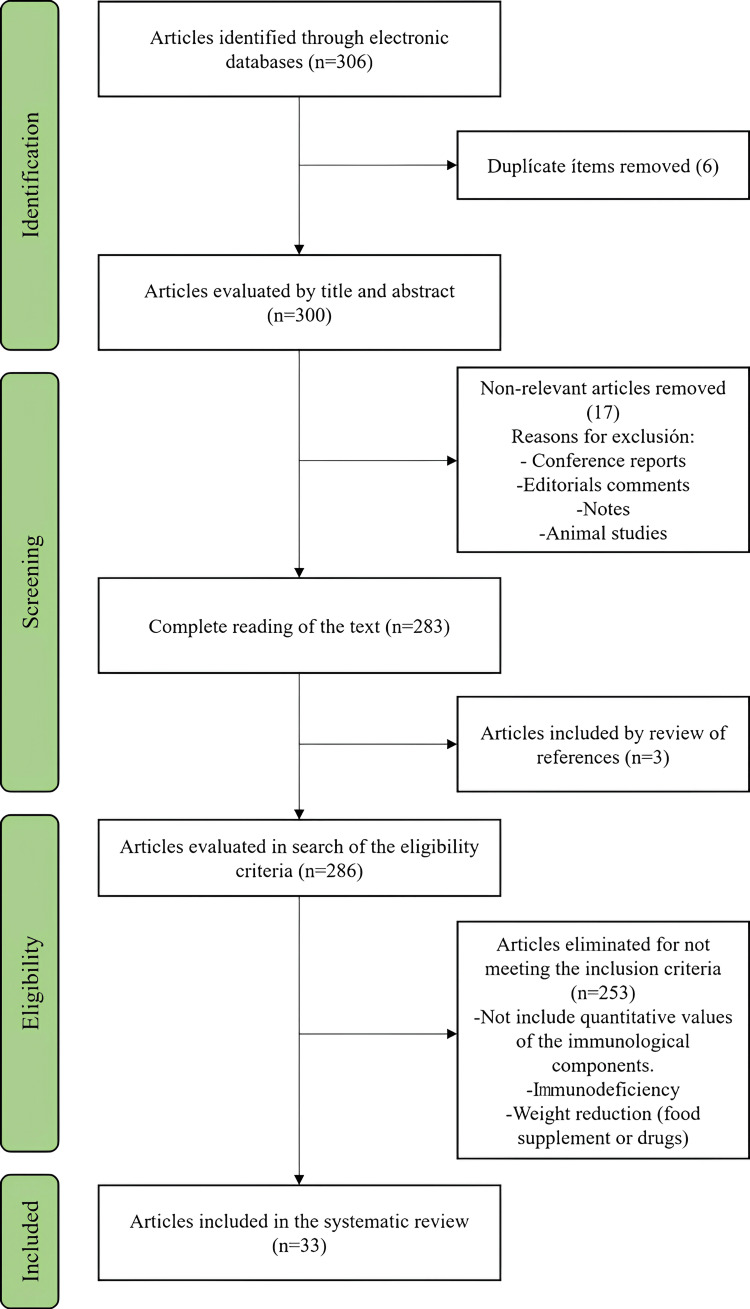
Flowchart of search, review, and inclusion of articles in the systematic review

A systematic search for changes in the immunological components of breast milk in obesity was carried out through search engines and specialized databases, such as Academic Google, PubMed, and Scopus. Thesauri breast feeding, obesity, immunology, and human milk in English and Spanish were used. Those articles published from January 2000 to October 2022 were selected for the first round. No AI program was used in the search or analysis of the articles. Articles that were conference reports, editorials, comments, notes, and animal studies were discarded in the first evaluation. Subsequently, those studies that did not include quantitative values of the immunological components were excluded, and those in which the values were reported as a percentage or interval were eliminated, as well as those in which the analysis of breast milk was not the main objective of the study or if the study group had any type of immunodeficiency or they were not articles from original research. Of those articles in which there was an intervention related to weight reduction or the use of a food supplement or drug, only data from the control group were taken. The PRISMA 2020 guides were used to build this article, and a total of 306 articles were reviewed, of which a total of 33 were included in the final review (Figure 1). To provide an order to the results, the components of breast milk are described, followed by the modifications described in obesity.

Results

Articles of Comparison Between Adequate Weight and Overweight

The results of the included articles are summarized in Tables 1, 2, constructed using various articles that compare immunological factors, such as signaling molecules [45-48], Ig [43,48], complement factors [48], other enzymes of immunological use [49], and microbiota [46]. In these, a relative elevation of the various pro-inflammatory factors and a decrease in regulatory ones were observed. However, in various articles, these differences are not statistically significant, and it is important to mention that even the microbiome is altered in overweight and obesity.

**Table 2:** Compilation of values of immunological factors between women with adequate weight and with obesity In the different articles selected, the mean or median was included (depending on which was used in the article). Therefore, according to the statistic used, it was accompanied by the standard deviation or the interquartile range (IQR). This was only possible in those articles that included it. In relation to the pathological state, the one that marked the greatest difference in weight, i.e., obesity, was chosen from each article, except in those in which this was not possible. ^nd^Statistical analysis was not possible due to the number of samples. ^a^Statistical differences were calculated using the Mann–Whitney U-test. ^b^Statistical differences in bacterial prevalences between BMI groups (normal and obese) were calculated using the χ^2^ test (2 × 2). EGF, epithelioid growth factor; IGF-1, insulin-like growth factor; CRP, C-reactive protein

Variable	Sample			Adequate weight (BMI > 19 Kg/m^2^ y < 25 Kg/m^2^)	Obesity and/or overweight, depending on the reference (IMC ≥ 25 Kg/m^2^)	p-value	Reference
Interleukin							
TNF-α (pg/mL)	Colostrum			10.58	12.91	0.7353	Fujimori et al. [45]
	Colostrum			9.87	11.4	0.560	Collado et al. [46]
	Mature			10.6	10.23	0.770	
TGF-β (pg/mL)	Colostrum			2 247.06	1 300.67	0.094	Collado et al. [46]
	Mature			1 006.03	550	0.188	
INF-γ (pg/mL)	Colostrum			122.6	141.91	0.566	Collado et al. [46]
	Mature			201.57	187	0.775	
sCD14 (µg/mL)	Colostrum			28.22	23.21	0.275	Collado et al. [46]
	Mature			5.54	4.35	0.210	
IL-2 (pg/mL)	Colostrum			29.3	29.4	0.987	Collado et al. [46]
	Mature			21	26.38	0.370	
IL-4 (pg/mL)	Colostrum			18.23	20.8	0.604	Collado et al. [46]
	Mature			20.74	20.34	0.941	
IL-6 (pg/mL)	Colostrum			62.86	81.85	0.381	Collado et al. [46]
	Mature			22.12	13.22	0.107	
IL-10 (pg/mL)	Colostrum			8.8	11.35	0.330	Collado et al. [46]
	Mature			11.46	11.8	0.915	
EGF (ng/mL)	Not reported	Median		0.038	0.040	0.013	Khodabakhshi et al. [47]
		IQR	Lower limit	0.037	0.038		
			Upper limit	0.039	0.045		
IGF-1 (ng/mL)	Not reported	Median		89.63	75.09	0.787	Khodabakhshi et al. [47]
		IQR	Lower limit	64.3	55.35		
			Upper limit	104.79	117.41		
CRP (mg/mL)				4		0.002	Fujimori et al. [48]
	Colostrum	Median			6 mg/mL		
		IQR	Lower limit	0	0		
			Upper limit	8	12		
Breast microbiota profile							
Total bacteria (log gene copy number/mL)	Colostrum	Prevalence (%)		100%	100%	0.024^a^	Collado et al. [46]
		Median		5.9	6.18	nd^b^	
		IQR	Lower limit	5.39	6		
			Upper limit	6.26	6.35		
	Mature	Prevalence (%)		100	100	0.700^a^	
		Median		6	6.14	nd^b^	
		IQR	Lower limit	5.68	5.75		
			Upper limit	6.31	6.26		
Bifidobacteria group (log number of gene copies/mL)	Colostrum	Prevalence (%)		100	85	0.462^a^	Collado et al. [46]
		Median		5.72 I	5.46	0.054^b^	
		IQR	Lower limit	5.39	4.57		
			Upper limit	6.02	5.83		
	Mature	Prevalence (%)		100	88.2	0.009^a^	
		Median		5.86	5.3	0.068^b^	
		IQR	Lower limit	5.37	4.63		
			Upper limit	6	5.84		
*Staphylococcus* group (log gene copy number/mL)	Colostrum	Prevalence (%)		56.5	85	0.025^a^	Collado et al. [46]
		Median		4.63	5.25	0.043^b^	
		IQR	Lower limit	4.21	4.25		
			Upper limit	5.11	5.72		
	Mature	Prevalence (%)		77.8	100	0.023ª	
		Median		4.4	4.94	0.036^b^	
		IQR	Lower limit	4.2	4.23		
			Upper limit	5.05	5.57		
*Staphylococcus aureus* (log gene copy number/mL)	Colostrum	Prevalence (%)		39.1	45	nd^a^	Collado et al. [46]
		Median		4.89	4.76	0.697^b^	
		IQR	Lower limit	4.46	4.53		
			Upper limit	5.13	5.72		
	Mature	Prevalence (%)		62.9	60.6	0.050^a^	
		Median		4.79	4	0.603^b^	
		IQR	Lower limit	4.35	3		
			Upper limit	5.28	4.74		
*Akkermansia muciniphila* (log gene copy number/mL)	Colostrum	Prevalence (%)		47.8	45	0.711ª	Collado et al. [46]
		Median		2.28	4.76	0.425^b^	
		IQR	Lower limit	1.71	4.53		
			Upper limit	3.27	4.95		
	Mature	Prevalence (%)		22.2	47	nd^a^	
		Median		2.37	2.88	0.085^b^	
		IQR	Lower limit	1.73	1.65		
			Upper limit	3.11	3.11		
*Lactobacillius* group (log number of gene copies/mL)	Colostrum	Prevalence (%)		91.3	95	0.003^a^	Collado et al. [46]
		Median		5.83	6.45	0.635^b^	
		IQR	Lower limit	4.89	6		
			Upper limit	6.3	6.71		
	Mature	Prevalence (%)		95.6	88.2	0.791^a^	
		Median		6.02	6.14	0.624^b^	
		IQR	Lower limit	5.29	5.85		
			Upper limit	6.34	6.36		
*Enterococcus* group (log gene copy number/mL)	Colostrum	Prevalence (%)		91.3	95	0.103^a^	Collado et al. [46]
		Median		4.38	4.6	0.635^b^	
		IQR	Lower limit	3.89	4.11		
			Upper limit	4.52	4.86		
	Mature	Prevalence (%)		84.2	82.3	0.154^a^	
		Median		4.29	3.86	0.297^b^	
		IQR	Lower limit	4.48	3.37		
			Upper limit	4.45	4.35		
*Clostridium coccoides* (log gene copy number/mL)	Colostrum	Prevalence (%)		39.1	65	nd^a^	Collado et al. [46]
		Median		4.94	4.91	0.091^b^	
		IQR	Lower limit	4.25	4.37		
			Upper limit	5.3	5.26		
	Mature	Prevalence (%)		66.7	76.5	0.751ª	
		Median		5.36	4.94	0.488^b^	
		IQR	Lower limit	4.7	4.29		
			Upper limit	5.55	5.44		
*Streptococcus* (log gene copy number/mL)	Colostrum	Prevalence (%)		100	95	0.387^a^	Collado et al. [46]
		Median		3.77	3.84	0.278^b^	
		IQR	Lower limit	3.47	3.72		
			Upper limit	3.88	4.02		
	Mature	Prevalence (%)		100	94.1	0.122^a^	
		Median		3.64	3.79	0.202^b^	
		IQR	Lower limit	3.32	3.51		
			Upper limit	3.81	4.16		
Inmunoglobulins							
IgA (g/L)	Colostrum	Mean		5.67	5.6		Islam et al. [43]
		Standard deviation		1.65	1.47		
	Colostrum	Median		3.3	5.1	0.001	Fujimori et al. [48]
		IQR	Lower limit	2.3	3.3		
			Upper limit	5.5	9.6		
IgM (g/L)	Colostrum	Mean		0.47	0.47		Islam et al. [43]
		Standard deviation		0.09	0.01		
	Colostrum	Median		1.3	1.4	0.825	Fujimori et al. [48]
		IQR	Lower limit	0.85	1		
			Upper limit	2.2	3.4		
IgG (g/L)	Colostrum	Mean		0.096	0.093		Islam et al. [43]
		Standard deviation		0.024	0.02		
	Colostrum	Median		0.4	0.4	0.947	Fujimori et al. [48]
		IQR	Lower limit	0.1	0.2		
			Upper limit	0.6	0.6		
Other antimicrobial proteins and enzymes							
Lactoferrin (mg/mL)	Colostrum	Mean		2.89	3.49	0.27	Houghton et al. [49]
		Standard deviation		0.27	0.24		
	Transition	Mean		0.66	1.42	0.14	
		Standard deviation		0.14	0.16		
C3 (mg/dL)	Colostrum	Median		91.7	95.7	0.001	Fujimori et al. [48]
		IQR	Lower limit	50.5	41.3		
			Upper limit	110.2	130.9		
C4 (mg/dL)	Colostrum	Median		30.1	28.8	0.040	Fujimori et al. [48]
		IQR	Lower limit	15.6	17.7		
			Upper limit	34.8	40.2		

Discussion

Breastfeeding is an indispensable factor in the life of the baby and also continues with the close relationship between mother and child after birth. Colostrum (early milk produced up to days 4-5 postpartum) and mature milk contain many components that increase the infant’s resistance to infection [50]. Based on the selected articles, abnormally high levels of some immunological profile parameters were observed in the milk of obese mothers. In colostrum, pro-inflammatory ILs such as TNF-α, IFN-γ, and IL-6 stood out. IFN-γ was found to increase the Th1/inflammatory response and suppress the Th2/allergic response [30]. TNF-α triggers an inflammatory response together with IL-6, which is a proinflammatory cytokine. It should be noted that levels drop when transitioning to mature breast milk. High values of CRP were observed even after the transition from colostrum to mature milk, which is a marker of active inflammation from the mother in the context of chronic inflammation due to obesity.

Overweight women showed lower levels of e-TGF-β2, insulin-like growth factor 1 (IGF-1), and soluble CD14 (sCD14, a marker of macrophage activation) in breast milk than normal-weight mothers. TGF-β regulates inflammation and wound repair and prevents allergic diseases [50]. Milk-borne IGF-1 acts as a growth factor for intestinal maturation, and sCD14 is a myeloid differentiation antigen produced mainly by monocytes and macrophages. Human breast milk contains high concentrations of sCD14, which is derived from the epithelial cells of the mammary glands. This molecule is involved in innate immune responses and may control homeostasis mechanisms in the neonatal gut. Ingesting milk containing sCD14 can prevent excessive immune reactivity and tissue damage found in neonatal pathologies [34]. All these functions can be inhibited or diminished by having low values and can affect the health of the baby.

In addition, higher levels of bacteria from the *Staphylococcus* group and lower levels of bacteria from the *Bifidobacterium* group were detected in overweight mothers compared to normal-weight mothers. The prevalence of *Akkermansia muciniphila*-like bacteria was also higher in overweight mothers, and the number of these bacteria was related to IL-6 concentration in colostrum [42]. There is evidence that a reduced level of the anti-inflammatory cytokines IL-10 and IL-4 and an elevated level of the pro-inflammatory cytokines TNF-α and IFN-γ were associated with an elevated level of *A. muciniphila* [50].

There are levels that were not greatly affected or remained normal in obese mothers, such as EGF, IL-2, IL-4, and IL-10. IL-10 is a regulator of immune function and prevents inflammation. Epithelioid growth factor decreases the expression of proinflammatory cytokines.

The concentration of Ig in breast milk was similar in mothers with normal weight and mothers with obesity. Human milk provides the only source of sIgA during the first four weeks of life due to the lack of functional plasma cells in the infant. sIgA comprises up to 80-90% of the Ig present in breast milk with a higher concentration in colostrum and in the breast milk of mothers who give birth prematurely [27]. In contrast, other important Ig (IgM) isotypes, whose roles include promoting inflammation, are present in modest or very low concentrations. IgG is produced in the mammary gland as well as crosses the placenta and is detected in most colostrum samples from mothers, adding to the immune protection the baby needs.

In relation to antimicrobial proteins and enzymes, it was found that the levels of lactoferrin and C3 showed a slight increase in obese mothers, which may favor the baby by eliminating the trigger of the acute inflammatory response, reducing the expression of proinflammatory cytokines and inflammation, in addition, to regulate the intestinal microbiome. Complement proteins C3 and C4 help eliminate non-beneficial bacteria.

The limitations of this review are related to the limited number of studies on the immunological alterations of breast milk, further reducing those that investigate the effect on the newborn. In addition, the available evidence is limited to cross-sectional studies and a few experimental or observational studies with a high level of evidence (cohort or case-control studies), which in turn limits the level of evidence they can provide.

The axis of this work is to encourage future research to address this issue in a more in-depth way and through designs that provide more solid evidence on the possible effects on offspring, as well as including larger sample sizes so that what is identified can be extrapolated to the population and better serve to establish population health measures based on the best available evidence.

## Conclusions

Human milk is the ideal form of nutrition for the newborn. Regulatory cytokines prevent the activation of proinflammatory signaling pathways and control them once activated. Antimicrobial factors, such as lactoferrin and complement factors, kill pathogens directly. The data obtained in this review support the hypothesis that the metabolic changes promoted by obesity can alter the biochemical and immunological parameters of breast milk, with an increase in cytokines and the presence of immune cells and a decrease in important factors, such as TGF-β and IFG-1. However, we did not observe changes that would cast doubt on the protection that breastfeeding provides to newborns. A point to address in future studies is to verify the time in which the measurements of the components are made since this can influence the concentration of the milk components due to the behavior of the circadian rhythm.
